# Spatiotemporal Expression of SHH/GLI Signaling in Human Fetal Bladder Development

**DOI:** 10.3389/fped.2021.765255

**Published:** 2021-12-22

**Authors:** Haibao Zhang, Shan Xu, Dalin He, Xinyang Wang, Guodong Zhu

**Affiliations:** ^1^Department of Urology, The First Affiliated Hospital of Xi'an Jiaotong University, Xi'an, China; ^2^Oncology Research Lab, Key Laboratory of Environment and Genes Related to Diseases, Ministry of Education, Xi'an, China; ^3^Key Laboratory for Tumor Precision Medicine of Shaanxi Province, Xi'an Jiaotong University, Xi'an, China

**Keywords:** bladder development, sonic hedgehog signaling, GLI1, smoothened (SMO), patched

## Abstract

**Objectives:** Sonic hedgehog (SHH) signaling is important in bladder development. Mice with defective hedgehog signaling develop bladder anomalies. Clinically, urinary tract malformations are reported in human fetuses and infants with mutations of SHH and related signaling pathway genes. Information on the expression of SHH and associated signaling genes in normal human bladder development is fragmentary. This study determined the temporal and spatial expression patterns of SHH signaling pathway components in human fetal bladders by immunohistochemistry (IHC).

**Material and Methods:** Twenty-four bladder specimens from 16 male and 8 female human fetuses aged 12- to 36-week (wk) were obtained from the First Affiliated Hospital of Xi'an Jiaotong University. The tissue slides were processed for IHC staining with SHH, Patched1 (PTC-1), Patched2 (PTC-2), Smoothened (SMO), GLI1 and proliferating cell nuclear antigen (PCNA). The expression levels of each gene were analyzed by semi-quantitative histological scoring system.

**Results:** High intensity of SHH and SMO expression was detected in developing bladder urothelial cells, with no staining in lamina propria (LP), but with minimal expression of SMO in differentiating smooth muscle (SM) layers. The spatial distribution pattern of PTC1 and GLI1 was more complex with minimal expression in the LP layer, moderate expression in the SM layer, and high expression in the urothelium. PTC2 expression was mainly localized in the urothelium and LP, but no expression in the SM layer. All of the SHH signaling components were detected in fetal bladder tissues throughout the development, with expression peaks at 12- and 23-wk, coinciding with high cell proliferation as indicated by PCNA staining in the cell nuclei of urothelium and SM.

**Conclusions:** The autocrine SHH signaling in the developing urothelium, and paracrine SHH signaling in the developing smooth muscle layer, mediated by SMO, PTC-1 and GLI1 were demonstrated during human bladder development. Expression of SHH signaling components peaked at 12-and 23-wk. The first expression peak at 12-wk may relate to urothelium growth, SM induction, and dilation of the bladder cavity. The second expression peaked at 23-wk may relate to urothelium and SM layer differentiation.

## Introduction

The human urinary system originates from the intermediate mesoderm of the developing embryo, and bladder is one of the most important organs in human body for collecting and storing urine. Human bladder and ureterovesical junction develop from urogenital sinus (UGS) and derived from the hindgut during the fourth to seventh weeks of gestation (1, 2). The hindgut terminates as the cloaca, from which the UGS epithelium differentiates into bladder urothelium, and the UGS mesenchyme differentiates into smooth muscle (SM) and the lamina propria. SM is a crucial component of bladder tissue, and many epithelial as well as mesenchymal related signals are necessary for bladder SM development (3, 4). Without the stimulation of epithelium, the UGS mesenchyme cannot differentiate into SM.

Sonic hedgehog (SHH) is widely studied among three types of hedgehog homologs in human body, and it is related to many biological phenomena. Previous studies have demonstrated that SHH, transforming growth factor-β (TGF-β), bone morphogenetic protein 4 (BMP4), and fibroblast growth factor receptor 2 (FGFR2) are the key signaling factors in bladder development, and they are interconnected in a complicated regulatory network. Some animal experiments have shown that a certain concentration of SHH is particularly indispensable for the development of bladder musculature and its function (5, 6). SHH signaling has been implicated in the development of neural tissues, craniofacial skeleton, hair, teeth, lung, kidney, gastrointestinal tract, and prostate (7). Similarly, SHH signalings contribute to bladder development during embryogenesis, but it can also impact bladder tumorigenesis and cancer stemness in adulthood (8).

Previous studies based on animals have shown that SHH and its downstream signaling molecules are involved in bladder development, relevant study (6) has identified the function of SHH/PTC-1/GLI2/BMP4 pathway, SHH binds to PTC-1 and induces the formation of bladder smooth muscle through activating Gli2, thereby up-regulating BMP4 and positively affecting the proliferation of mesenchymal cells. Numerous animal studies (9, 10) have shown that classical SHH pathway involves in multiple biological activities of bladder, including cell fate determination, embryo morphogenesis, tissue differentiation, and programmed cell death.

However, it is not easy to acquire normal human developing bladder specimens, and results from mice or *in vitro* studies cannot accurately represent human beings. The expression of SHH signalings in different stages of normal human bladder development is largely unknown. In this study, we determined the temporal and spatial expression patterns of SHH signaling components in the developing human fetal bladder tissue samples by immunohistochemistry, and we explored the possible role of SHH signaling in the formation and development of human urinary bladder.

## Materials and Methods

### Specimen Collection and Preparation

Twenty-four fetal bladder specimens were obtained with the approval of the Institutional Review Board of the First Affiliated Hospital of Xi'an Jiaotong University. Informed consent was obtained by the consulting obstetricians, and all specimens used in this study were considered discarded tissues with devoid of any identifiers. Gestational age was determined from the date of the last menstrual period and was confirmed with crown-rump length and body length measurements. The gestational age distribution and the total number of tissue specimens (in parenthesis) were: 12 weeks (2 cases), 16 weeks (2 cases), 18 weeks (2 cases), 21 weeks (2 cases), 23 weeks (2 cases), 24 weeks (2 cases), 26 weeks (2 cases), 28 weeks (2 cases), 31 weeks (2 cases), 32 weeks (2 cases), 33 weeks (2 case), 36 weeks (2 cases). All those fresh fetal bladder specimens were taken and fixed in 4% paraformaldehyde for 24 h at least, embedded in paraffin, and sliced at 4 μm thickness for immunohistochemical study.

### Primary Reagents

Mouse anti-human SHH (BD-610182) primary antibody was purchased from American BD company, mouse anti-human SMO (SC-8424), PCNA (SC-6260), GLI1 (SC-8598), PTC-1 (SC-3039), PTC-2 (SC-4853) primary antibody was purchased from Santa Cruz, USA, and immunohistochemistry kit was purchased from Dako, USA.

### Immunohistochemistry Staining

The EnVision two-step method was used for immunohistochemistry staining, and the experimental procedure was carried out according to the kit instructions. The tissue slides were dewaxed by xylene, hydrated by gradient ethanol (100-30%), rinsed with distilled water for 3 min, and then placed in 0.01 mol/L sodium citrate buffer (pH 6.0) and microwaved for 5 min for antigen retrieval, and naturally cooled at room temperature. The slides were washed with 0.01 mol/L PBS (pH 7.4) for three times, and subjected to Dual Endogenous Enzyme Block for 10 min. After washing in 0.01 M PBS (pH 7.4) for three times, the slides were incubated overnight with the diluted primary antibody or the PBS solution as the negative control. After washing with 0.01 mol/L PBS (pH 7.4) for three times, the slides were subjected to 30 min DakoCytomation EnVision+ HRP reagent incubation for anti-mouse and anti-rabbit secondary antibodies, or 15 min Biotinylated Link coupled with 15 min Streptavidin-HRP incubation for goat antibodies. Signals were detected by adding substrate hydrogen peroxide using diaminobenzidine as a chromogen and followed by 2 min hematoxylin counterstaining. In the end, all the slides were routinely dehydrated, transparence, sealed and observed under Nikon Eclipse 600 Microscope (Nikon, Tokyo, Japan).

### Scoring of Immunohistochemical Staining

All of the immunostained slides were analyzed by two histopathologists, and eight fields in each slide were randomly selected and examined under a 40× objective lens of the microscope. All fields were assessed using a semiquantitative histological scoring system (H-score) described previously (11). In each field, the staining intensity (I) was graded as 0–no staining, 1–weak, 2–moderate, and 3–strong and the proportion (P) of cells with the observed intensity was recorded (from 0–none to 1.0–the entire population of examined cells). H-score for each field was determined by the sum of all I×P products.

## Results

### Expression of SHH, SMO, PCNA in Fetal Bladder Tissues at Different Gestational Weeks

**Figure 1:**
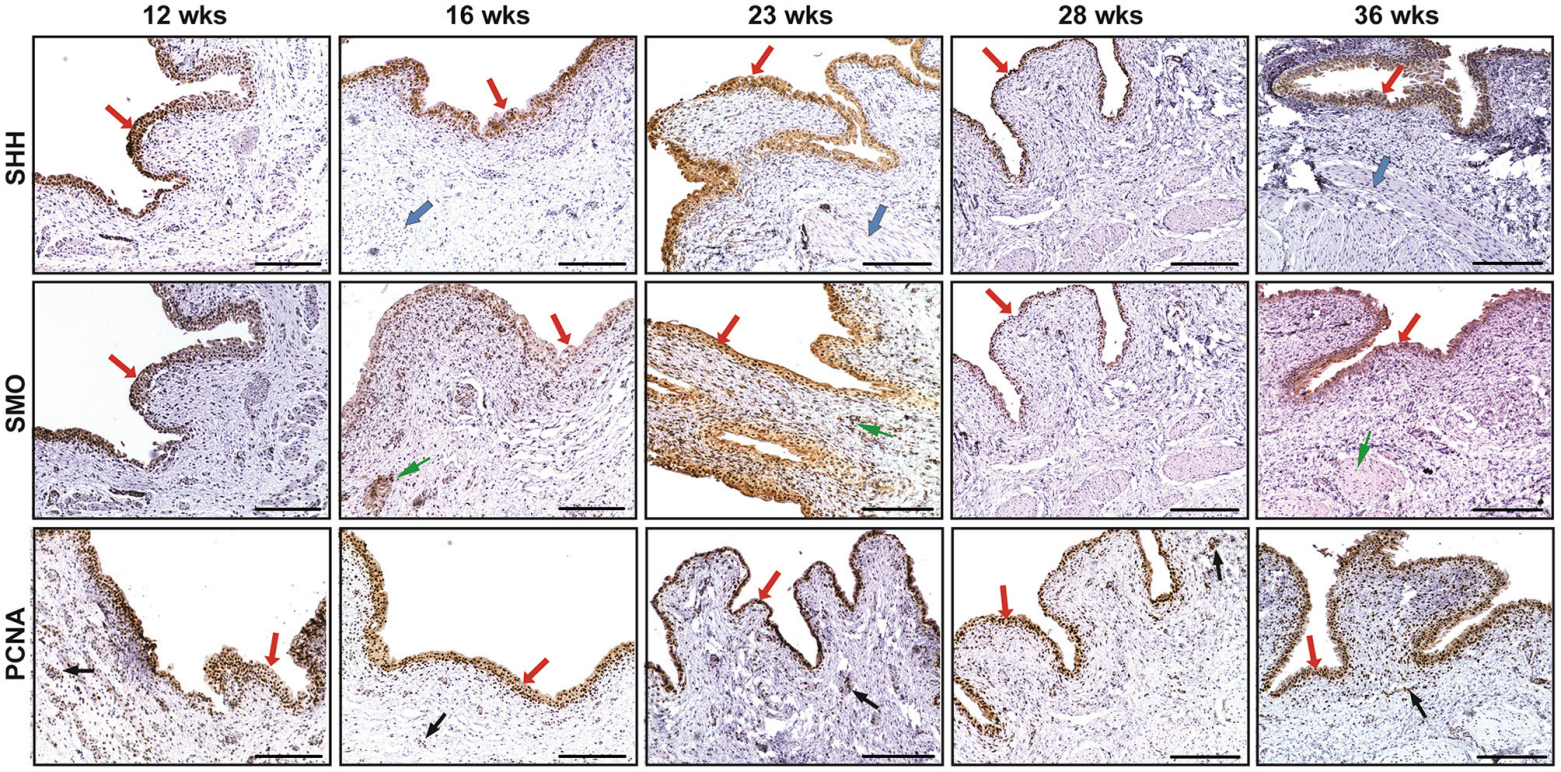
The immunohistochemical staining of sonic hedgehog (SHH), smoothened (SMO), and proliferating cell nuclear antigen (PCNA) in human fetal bladders at different gestational weeks (wks). The expression of SHH and SMO was mainly detected in the developing bladder urothelial cells (see RED arrows), but not in the lamina propria layer. The expression of SHH could not be examined in the differentiating smooth muscle layer (see BLUE arrows); however, the minimal expression of SMO could be detected in them (see GREEN arrows). The expression of PCNA could be detected in the nucleus of the developing urothelium (see RED arrows) and mesenchymal cells (see **BLACK** arrows) of the fetal bladder with different expression levels. Scale bars: 100 μm.

SHH were mainly expressed in the fetal bladder urothelial cells but not in the lamina propria and differentiating smooth muscle layers. The expression of SMO could be largely detected in the developing bladder urothelium with minimal staining in the mesenchymal cells of the fetal bladder smooth muscle layers. However, the expression of PCNA could be detected in the nucleus of the developing urothelium and mesenchymal cells of the fetal bladder with stronger intensity than that of SHH and SMO (Figure 1). With the increase of gestational age from 12 to 36 weeks, the fetal bladder urothelial and the smooth muscle layers were gradually formed and differentiated, and the expression level of SHH, SMO and PCNA formed two peaks at 12 weeks and 23 weeks, respectively (**Figure 3**).

### Expression of PTC-1, PTC-2, and GLI1 in Fetal Bladder Tissues at Different Gestational Weeks

**Figure 2:**
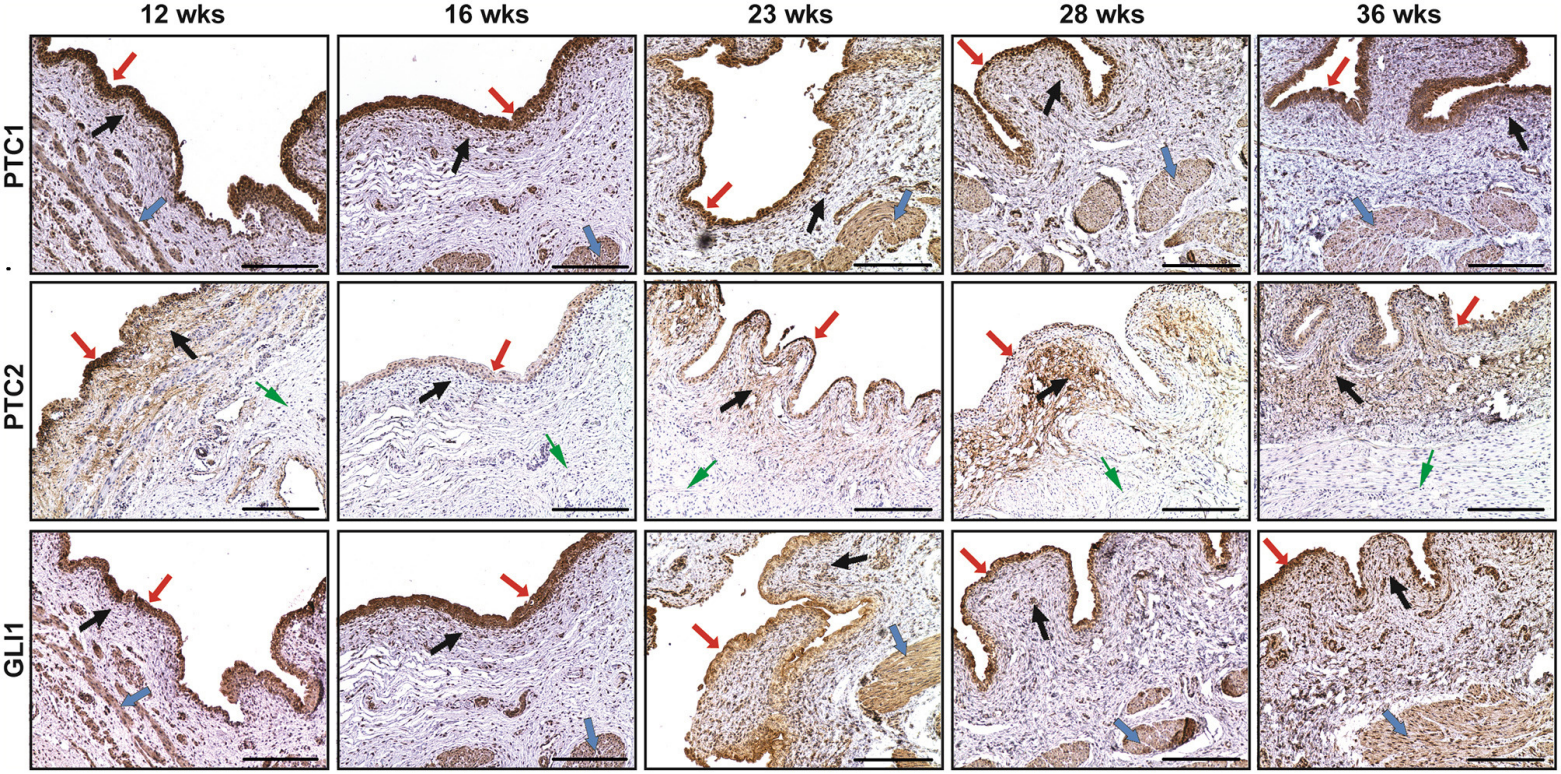
The immunohistochemical staining of patched-1 (PTC1), patched-2 (PTC2), and GLI1 in human fetal bladders at different gestations. The spatial distribution patterns of PTC1 and GLI1 were characterized by minimal expression in the lamina propria (**BLACK** arrows), moderate expression in the smooth muscle layer (BLUE arrows), and high expression in the urothelium (RED arrows). PTC2 expression was mainly localized in the urothelium (RED arrows) and lamina propria (**BLACK** arrows), but not in the smooth muscle layer (GREEN arrows). Scale bars: 100 μm.

**Figure 3:**
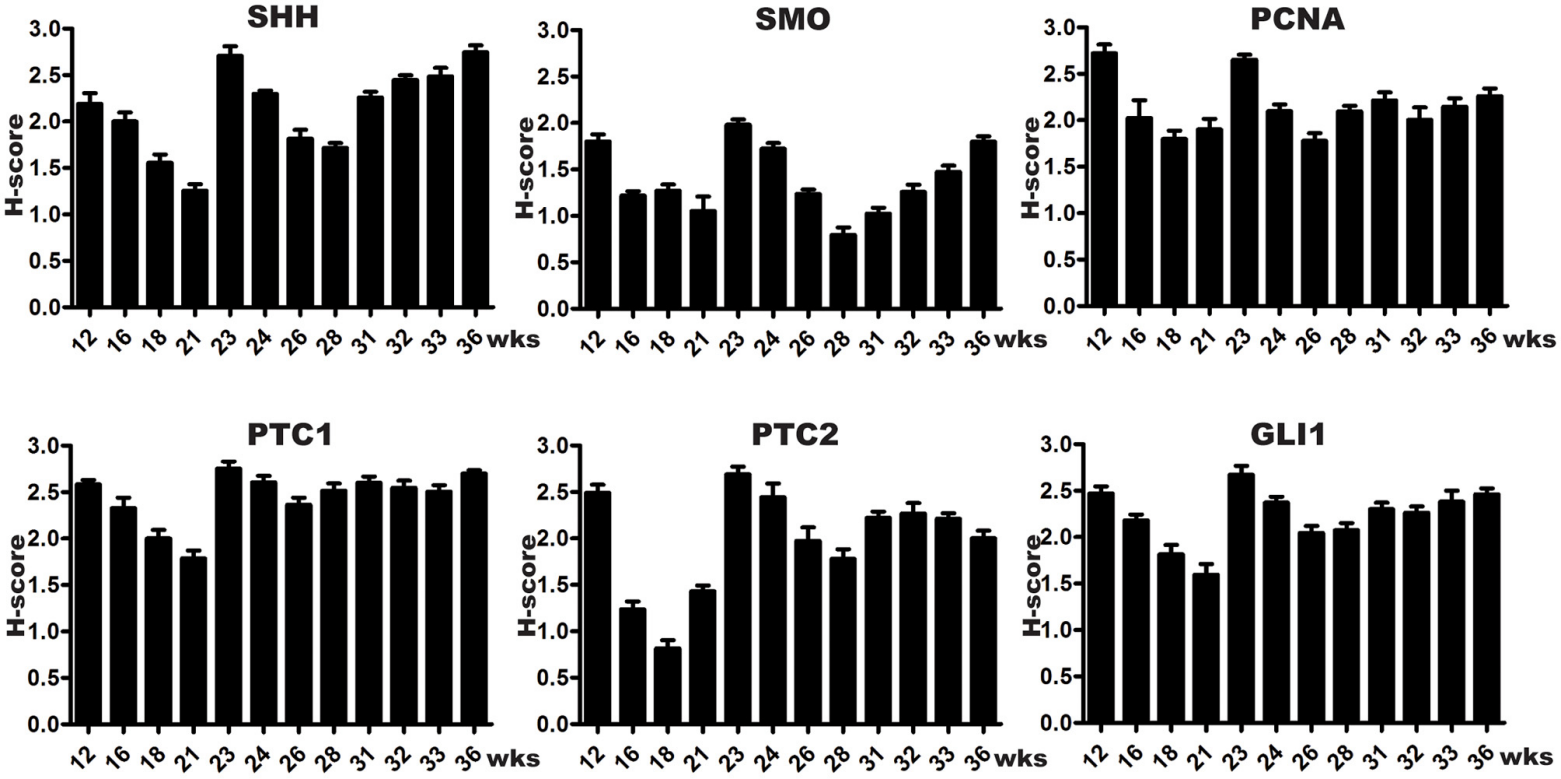
Semi-quantitative histological scoring system (H-scores) of SHH signaling components and PCNA expression in developing human bladders. Every column indicates two samples, and each bar represents the mean SEM (standard error of mean) of H-scores derived from tissue specimens at a specific gestational week. All SHH signaling components were detected in fetal bladder tissues throughout the development, with expression peaks at 12 and 23 weeks (wks), coinciding with high cell proliferation as indicated by PCNA staining in the cell nuclei of developing urothelial and smooth muscle cells. The first expression peak at week 12 wks may relate to urothelium growth, smooth muscle cells induction, and dilation of the bladder cavity. The second expression peak at week 23 wks may relate to urothelium and smooth muscle layer differentiation.

The spatial distribution patterns of PTC1 and GLI1 were characterized by minimal expression in the lamina propria, moderate expression in the smooth muscle layer, and high expression in the urothelium of the developing bladder. The expression of PTC2 was mainly localized in the urothelium and lamina propria, but not in the smooth muscle layer (Figure 2). With the developmental process, the temporal expression patterns of PTC-1, PTC-2, and GLI1 showed two expression level peaks, which could be found in 12 and 23 weeks, respectively (Figure 3).

## Discussion

Normal human bladder development requires two indispensable aspects, including the precise temporospatial distribution of key morphogenetic and regulatory signals to generate radial patterning for the subsequent compartmentalization of distinct cellular and tissular phenotypes; on the other hand, it is highly depending on the cross-talk between urothelium and its surrounding mesenchyme, which is called epithelium-mesenchyma interaction. Tissue recombination experiments have proved that bladder urothelial epithelium is required to form mesenchyme and differentiate into smooth muscle through diffused signaling molecules in cellular microenvironment (12).

The expression of SHH in the early development of organs has their temporal and spatial specificity, and it gives rise to regulating the morphogenesis of organs through autocrine and paracrine effects on both epithelial tissues and surrounding mesenchymal cells (13–16); however, the abnormal expression of SHH is also the cause of many congenital malformations. Although the SHH/GLI axis has been argued to play a crucial role in bladder urothelium induction and smooth muscle differentiation, its precise role in overall patterning and organogenesis remains to be determined (15, 17). Secreted protein SHH binds to the PTC receptor, releasing the inhibition on SMO and modulating transcription factor GLI complex. SHH at different concentrations are selective for distinct position of PTC expressing cells, together with the released M-phase promoting factor (MPF) and induce cellular proliferation via regulating cyclinB1/Cdc2 complex positively; activated GLI2 can translocate into the nucleus to up-regulate the expression of BMP4, ultimately can pattern and differentiate bladder smooth muscle by activating TGF-β/Smad signaling (18).

Previous studies (6, 7, 19) have proved SHH signaling in the urogenital sinus where it acts as a promoter throughout bladder development, including establishing the cellular populations and smooth muscle formation, which is formed around 12 weeks of gestation in human (20); therefore, fetal bladder at 12 weeks of the gestational stages was chosen as the starting point in our study. Desouza et al. (21) characterized that SHH, PTC and GLI1 expressed in early urogenital system development in mice. Liu et al. (22) proved that PTC1 as well as GLI1 could be detected in the mesenchyme and epithelium before the induction of smooth muscle, and both of their expression level decreased subsequently in the mesenchyme after the differentiation of smooth muscle. The above studies implied that mesenchyme might be the precursor for the development of multiple urogenital organs and structures. Another study by Cheng et al. (23) characterized the expression of SHH, PTC, GLI-families in urogenital sinus at multiple developmental time points, and that SHH mutant mice manifest hypoplastic external genitalia, pelvic urethra and bladder development defect. As the downstream key components of SHH, the GLI-families play synergistic roles in regulating the patterns of bladder mesenchyme proliferation. Cheng et al. (6) used both wildtype and SHH/GLI knockout mice to characterize the SHH/GLI axis in detrusor smooth muscle formation, and they found that SHH^−/−^ and Gli2^−/−^ bladders were hypoplastic; and furthermore, SHH signaling could regulate bladder morphogenesis in a dose-dependent manner. In this study, we examined the SHH signaling components in normal human bladders, and no anatomical or histological abnormalities were observed. The above animal studies as well as transgenic mice have some similarities to our findings in human fetal bladders by using IHC examination. All of the studies indicated that SHH/GLI axis might regulate mesenchyme proliferation and differentiation.

Additionally, previous studies predicted that high levels of SHH and GLI2 expressing cells in the mesenchyme could support urothelial cell proliferation but suppress smooth muscle differentiation. Autocrine SHH through PTC could drive alterations of cell morphology but not relevant to GLI-mediated transcription (24). Alternatively, in the absence of SHH, PTC1 is proved to regulate G2/M checkpoint by dephosphorylating cyclin B1 to suppress cell mitosis (25). Meanwhile, SMO can also be regulated positively by PTC to promote cell proliferation (26). Similarly, we also found that the changes of SHH signaling at different time points were not completely consistent in this study. In one word, members of the SHH/GLI axis are interacting with each other and relatively independent at the appropriate time point, coordinating bladder cell proliferation and smooth muscle differentiation.

Tasian et al. (18) illuminated the difference between mice and human in terms of timing for bladder organogenesis. The bladder forms in the first trimester in humans while the third trimester in mice, in other words, animal experiments cannot epitomize human studies because of species differences. In this study, we collected specimens of normal human fetal bladder at successive time points, and provided a complete description of the expression of SHH/GLI axis in the process of human bladder development. However, some studies (27, 28) have suggested that abnormal expression of SHH is related to the formation of cancer stem cells through interacting with TGF-β1, which results in bladder tumorigenesis, even resistance to chemotherapy and radiotherapy. Therefore, based on the expression of SHH, making timely intervention may be one of the primary preventions for bladder cancer.

The autocrine action and paracrine action are two ways for cytokines playing biological effects, and both classical and non-classical hedgehog signal transduction pathways involve these two approaches. Many studies (5, 26, 29) held the opinion that SHH acted as an activated epithelial signal to promote mesenchymal proliferation in an autocrine way in mouse liver, kidney and bladder. Shiroyanagi et al. (9) proposed that SHH secreted by urothelium inducing smooth muscle differentiation, and they also performed *in vitro* studies to summarize that paracrine SHH from the urothelium regulates smooth muscle differentiation. Hence, we speculated that autocrine SHH in the developing urothelium, and paracrine SHH signaling in the developing smooth muscle layer, mediated by SMO, PTC1 and GLI1, were demonstrated during human bladder development.

Clinically, urinary tract malformations, such as ureteral duplication and ectopy, vesicoureteral reflux, bladder exstrophy are reported in human infants with mutations of SHH or its related signaling pathway genes. Birth defects are structural or functional abnormalities that existed before birth. Genetic counseling, prenatal screening and diagnosis are very important tools for the prevention and treatment for birth defects. With the development of medical technology, prenatal diagnosis can be made before birth of the fetus with a variety of advanced detection methods to explore the fetal development situations in utero, observing whether the fetus has malformation, making diagnosis of congenital diseases, and creating conditions for fetal intrauterine treatment or selective abortion. Between 18 and 24 weeks of gestation, amniocentesis, percutaneous umbilical cord blood puncture and fetal tissue biopsy can be safely performed, and then the molecular genetic examination can be carried out for the evaluation of possible developmental defects. Under the help of the identification of abnormal expression or mutations of some development related essential genes, such as SHH/GLI, the obstetricians might timely terminate the pregnancy with those problem genes to avoid of the possible birth defects, such as bladder exstrophy, or they can make full consideration for post-natal treatment or active intervention.

We would like to underline the limitations in this study. Firstly, we should have applied statistical tests to evaluate expression level of SHH signaling pathway components in fetal human bladder; however, it was inappropriate for studies with very small sample size, just like ours. Frankly speaking, fresh embryonic tissues with different gestational ages by induced labor were extremely difficult to obtain and to be used for experimental research. Hopefully, we are still going to stick with this study in the future, so as to look forward to more in-depth results when the sample size could be increased. Secondly, we did not examine the abundance of these signalings; however, once we collect more tissue samples in the future, we would like to use more accurate methods, such as western blot and quantitative RT-PCR, to analyze SHH signaling components at different stages of human bladder development and conduct the statistical analysis.

## Conclusion

In summary, autocrine SHH in the developing urothelium, and paracrine SHH signaling in the developing smooth muscle layer, mediated by SMO, PTC1 and GLI1, were demonstrated during human bladder development. The first expression peak of SHH signaling at 12 wks might relate to urothelium growth, smooth muscle cell induction, and the second expression peak of SHH signaling at 23 wks might relate to smooth muscle layer differentiation.

## Data Availability Statement

The original contributions presented in the study are included in the article/supplementary material, further inquiries can be directed to the corresponding author/s.

## Ethics Statement

The studies involving human participants were reviewed and approved by the Institutional Review Board of the First Affiliated Hospital of Xi'an Jiaotong University. The patients/participants provided their written informed consent to participate in this study.

## Author Contributions

GZ and DH conceived and designed this study. HZ and SX conducted specimen collections and all the immunohistochemical staining. XW implemented quality control. HZ and GZ wrote and revised the manuscript. All authors read and approved the final manuscript.

## Funding

This study was supported in part by the grants from the Fundamental Research Funds for the Central Universities of China (No. xjj2018zyts34) and the Research Funds on Social Development from the Department of Science and Technology of Shaanxi Province (No. 2020SF-119) to GZ.

## Conflict of Interest

The authors declare that the research was conducted in the absence of any commercial or financial relationships that could be construed as a potential conflict of interest.

## Publisher's Note

All claims expressed in this article are solely those of the authors and do not necessarily represent those of their affiliated organizations, or those of the publisher, the editors and the reviewers. Any product that may be evaluated in this article, or claim that may be made by its manufacturer, is not guaranteed or endorsed by the publisher.
